# Utility of support vector machine and decision tree to identify the prognosis of metformin poisoning in the United States: analysis of National Poisoning Data System

**DOI:** 10.1186/s40360-022-00588-0

**Published:** 2022-07-13

**Authors:** Omid Mehrpour, Farhad Saeedi, Christopher Hoyte, Foster Goss, Farshad M. Shirazi

**Affiliations:** 1 Data Science Institute, Southern Methodist University, Dallas, TX USA; 2 Rocky Mountain Poison & Drug Safety, Denver Health and Hospital Authority, Denver, CO USA; 3 Medical Toxicology and Drug Abuse Research Center (MTDRC), Birjand University of Medical Sciences (BUMS), Birjand, Iran; 4 Student Research Committee, Birjand University of Medical Sciences, Birjand, Iran; 5 University of Colorado Anschutz Medical Campus, Aurora, CO USA; 6 University of Colorado Hospital, Aurora, CO USA; 7 Department of Emergency Medicine, University of Colorado Hospital, Aurora, CO USA; 8 Arizona Poison & Drug Information Center, the University of Arizona, College of Pharmacy and University of Arizona, College of Medicine, Tucson, AZ USA

**Keywords:** Metformin, National Poison Data System, Support vector machine, Decision tree

## Abstract

**Background:**

With diabetes incidence growing globally and metformin still being the first-line for its treatment, metformin’s toxicity and overdose have been increasing. Hence, its mortality rate is increasing. For the first time, we aimed to study the efficacy of machine learning algorithms in predicting the outcome of metformin poisoning using two well-known classification methods, including support vector machine (SVM) and decision tree (DT).

**Methods:**

This study is a retrospective cohort study of National Poison Data System (NPDS) data, the largest data repository of poisoning cases in the United States. The SVM and DT algorithms were developed using training and test datasets. We also used precision-recall and ROC curves and Area Under the Curve value (AUC) for model evaluation.

**Results:**

Our model showed that acidosis, hypoglycemia, electrolyte abnormality, hypotension, elevated anion gap, elevated creatinine, tachycardia**,** and renal failure are the most important determinants in terms of outcome prediction of metformin poisoning. The average negative predictive value for the decision tree and SVM models was 92.30 and 93.30. The AUC of the ROC curve of the decision tree for major, minor, and moderate outcomes was 0.92, 0.92, and 0.89, respectively. While this figure of SVM model for major, minor, and moderate outcomes was 0.98, 0.90, and 0.82, respectively.

**Conclusions:**

In order to predict the prognosis of metformin poisoning, machine learning algorithms might help clinicians in the management and follow-up of metformin poisoning cases.

## Background

Diabetes has become a public health concern that gives rise to serious macrovascular and microvascular complications [1]. In 2015, there were approximately 415 million diabetes patients worldwide; this figure is expected to grow to over 600 million by 2040 [2]. Metformin is still the first-line therapy for diabetes among all medicines currently available [3]. However, even though metformin is a safe drug, poisoning can have a fatality rate of 30 to 50%, climbing to as much as 80% if done intentionally [4]. Due to the lack of an antidote and a conservative approach to managing metformin poisoning, early prognosis prediction based on initial presentation might be critical in diminishing the death rate. Some studies evaluated prognostic factors in metformin poisoning. These studies tried to emphasize the role of blood gas analysis in predicting the outcome [5, 6]. However, they reached different results. For example, Kajbaf et al. showed that the lactate concentration and mean arterial pH was not significantly different between non-survivors and survivors diagnosed with metformin poisoning [5]. On the other hand, Shojaei Arani et al. found that HCO3^−^ levels of less than 17.25 (mEq/L) and pH levels of less than 6.94 were associated with patient mortality among those with metformin poisoning [6]. This inconsistency might imply that using more sophisticated analysis such as AI might help physicians predict the outcome of metformin poisoning prognosis.

Medicine has paid much attention lately to new classification methods [7]. The fascinating part about machine learning techniques is that they can be taught how to assess medical risk predictions and simulate complicated clinical scenarios [8]. Thus, machine learning could boost precision medication delivery more than regression analysis [9]. Surprisingly, massive amounts of data heterogeneity can be combined using machine learning to uncover causal relationships between diseases and classify risk variables [10]. The decision tree (DT) examines the input data and shows the outcomes of the predicted relationships that have been identified as a consequence of the implementation of appropriate rules [11]. When dealing with huge amounts of variables and a limited sample size in medical research, the support vector machine (SVM) is another promising classification model [12]. SVM is a breakthrough tool that outputs findings while establishing a hyperplane, culminating in classification. Essentially, the underlying concept of SVM is to establish decision boundaries wherein characteristics are assessed concerning a hyperplane to demonstrate their significance. The attributes for which the hyperplane is drawn are called support vectors. Moreover, the distance between the hyperplane and the attributes is called margins. The optimal hyperplane is the one with the largest margin of support vectors.

The application of DT and SVM have been widely investigated among patients with drug overdose in recent times [13–15]. However, despite the serious outcomes, there has been no explicit application of machine learning techniques to study metformin overdose and provide a realistic strategy for determining the prognosis based on clinical and laboratory findings. Besides, although many of the important features of metformin poisoning are well known, machine learning algorithms to determine metformin poisoning are not studied yet. Artificial intelligence (AI) has been shown to help health care providers to diagnose diseases. Moreover, AI can aid physicians with their clinical decision-making and to predict the outcome [13, 16]. In addition, its performance in the field of medical toxicology received much attention recently [17–19]. However, it is important to understand how AI fits into large heterogeneous datasets and functions.

This study is the first study that used machine learning algorithms to predict metformin poisoning. We aimed to apply and test the decision tree and support vector machine models on a broad scale of patients with metformin overdose, taken from the National Poison Data System (NPDS), to evaluate the different categories of outcomes.

## Methods

### Study population and eligibility criteria

The data of this study was obtained from NPDS, which is the only real-time data repository of poisoning in the United States. American Association of Poison Control Centers (AAPCC) which maintains the NPDS, represents the 55 Poison Control Centers (PCCs). NPDS includes exposures to more than 400,000 substances that have been continuously reported by PCCs [20]. In addition, more than 2 million human exposure was reported to NPDS in 2019 [20]. Even though NPDS data does not contain all of the substance exposure in the country, every exposure reported to NPDS does not necessarily signify poisoning or toxicity. All metformin exposure cases reported to the NPDS between January 1, 2012, and December 31, 2017, were included in this study. However, we excluded those cases with missing data and duplicate ones. This study was not required Institutional review board approval based on the Colorado Multiple Institutional Review Board on Human Subjects Protection standards. All methods were carried out following relevant guidelines and regulations.

### Definition of terms

To develop our classification model, we defined some important features based on the NPDS guidelines as follows:

- Hypertension: Diastolic blood pressure greater than 90 mmHg or systolic blood pressure greater than 140 mmHg
- Hypotension: systolic blood pressure which is more than 15 mmHg less than usual systolic blood pressure that the patient has or less than 90 mmHg
- Elevated anion gap: Result of the following equation more than 12 mEq/L: [Na + − (Cl- + HCO3-)]
- Elevated creatinine: Creatinine level of more than 1.5 mg/dL or 133 μmol/L
- Tachycardia: Heart rate more than 100 beats per minute
- Renal failure: Acute and chronic renal failure that leads to clinically substantial loss of renal function and azotemia
- Electrolyte abnormality: Imbalance level of sodium, potassium, bicarbonate, chloride, calcium, magnesium, and phosphate
- Hypoglycemia: Glucose levels of less than 70 mg/dL or 3.9 mmol/L
- Acidosis: Bicarbonate level less than 20 mEq/L, pH less than 7.35, or elevated levels of lactic acid
- Minor outcomes: The minimal bothersome symptoms, including skin or mucous membrane manifestations.
- Moderate outcomes: The symptoms that are not life-threatening but are more prolonged than minor symptoms.
- Major outcomes: The life-threatening symptoms leading to significant complications.

### Development of classification model

First, the dataset was randomly divided into training (70%) and testing (30%) datasets. The prediction model was developed by utilizing a train set and then incorporating the various variables, including demographic data (age, sex), the purpose of exposure (suicidal, unintentional, etc.), chronicity, clinical features, etc. Next, the test set was utilized to evaluate the model performance to see how well it fits the training set. Every decision tree comprises some nodes, including root and leaf nodes and branches. The root node denotes the most important feature, whereas the leaf node depicts a decision by applying some IF-THEN rules. For example, the right and left directions indicate false and true when moving down the decision tree’s path. The evaluation of the decision tree model was performed through F-1 score, specificity, recall, precision, accuracy, and confusion matrix. Roc curves and precision-recall curves are provided for each model. Based on different probability thresholds, ROC curves illustrate the trade-off between true positive and false positive rates for a prediction model.

Using different probability thresholds, precision-recall curves summarize the trade-off between the true positive rate and positive predictive value of a predictive model. All the analyses were done in Python using the Sklearn library.

## Results

### Prognosis prediction based on decision tree

**Fig. 1:**
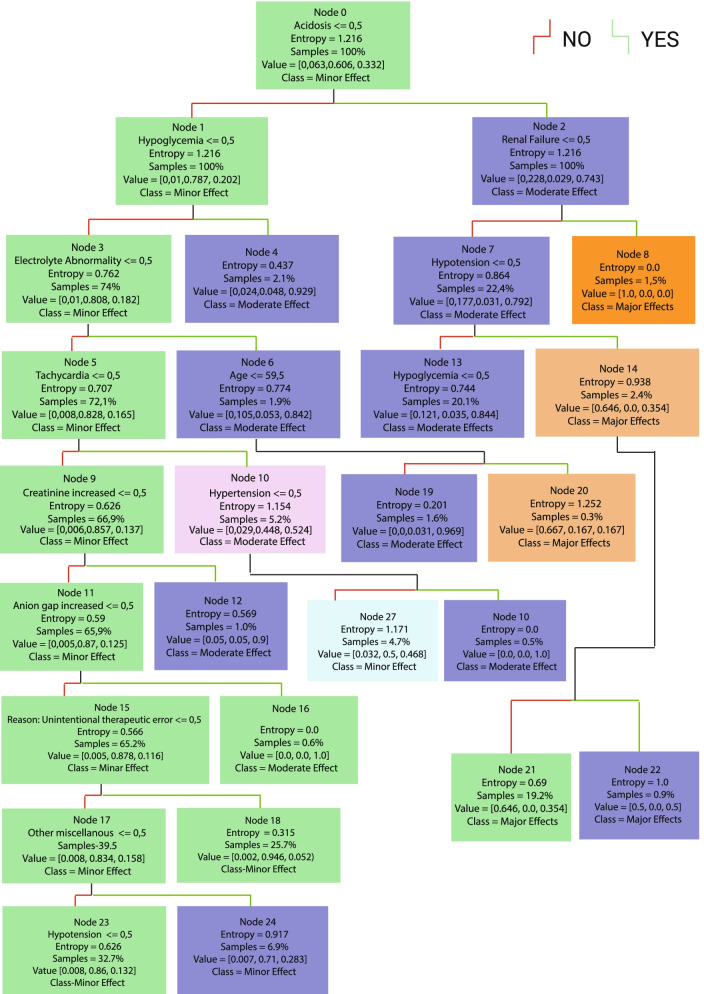
Decision tree model

**Fig. 2:**
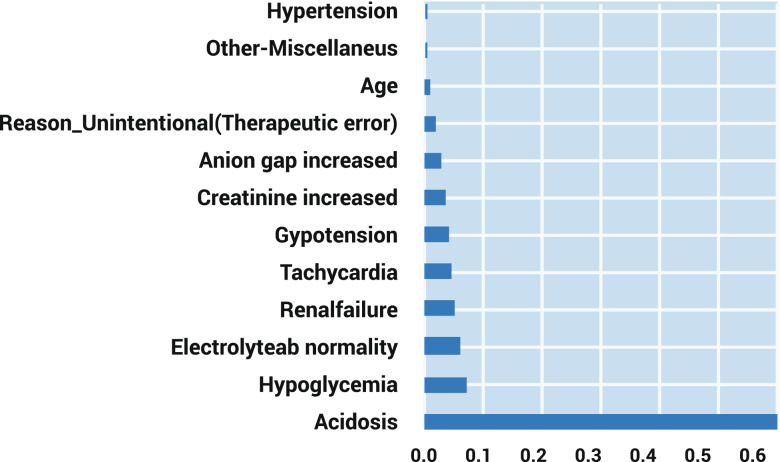
Important features based on decision tree algorithm

A total of 2878 cases with metformin exposure were included in this study. The decision tree model and 15 rules-driven from it are shown in Fig. 1 and Table 1, respectively. The most important feature of our model is shown in Fig. 2. Our decision model comprises 10 levels, 15 leaf nodes, and 29 nodes. Acidosis was the most determined prognostic factor, followed by hypoglycemia and electrolyte abnormality.

**Table 1:** The rules driven from the decision tree model

If acidosis and renal failure occur, patients will develop a major outcome (100%)	1
If acidosis and hypotension occur while renal failure does not occur, patients are more likely to develop major outcomes (64.6%)	2
If acidosis and hypoglycemia are present, while renal failure and hypotension do not occur, patients are more likely to develop major outcomes (50%)	3
If acidosis occurs while renal failure, hypotension and hypoglycemia do not occur, patients are more likely to develop moderate outcomes (86%)	4
If a patient experiences hypoglycemia without acidosis, moderate outcomes are more likely (92.9%)	5
If a patient with an age of more than 59.5 experiences electrolyte abnormalities without acidosis, hypoglycemia, the development of major outcomes is more likely (66.7%)	6
If a patient with an age of less than 59.5 experiences electrolyte abnormalities without acidosis, hypoglycemia, the development of a moderate outcome is very likely (96.9%)	7
If tachycardia and hypertension are present, while acidosis, hypoglycemia and electrolyte abnormalities are not present, patients develop moderate outcomes (100%)	8
If tachycardia is present without acidosis, hypoglycemia, electrolyte abnormalities and hypertension, patients are likely to develop a minor outcomes (50%)	9
If increased creatinine is present, while acidosis, hypoglycemia, electrolyte abnormalities and tachycardia are not present, patients are more likely to develop moderate outcomes (90%)	10
If an elevated anion gap is present, while acidosis, hypoglycemia, electrolyte abnormalities, tachycardia and elevated creatinine are not present, patients develop moderate outcomes (100%)	11
If a patient has an unintentional exposure without hypoglycemia, electrolyte abnormalities, tachycardia, elevated creatinine and elevated anion gap, the patient is more likely to develop minor outcomes (94.6%)	12
If acidosis does not occur, hypoglycemia does not occur, electrolyte abnormality does not exist, tachycardia does not exist, elevated creatinine does not exist, elevated anion gap does not exist, the reason for exposure is not unintentional, other miscellaneous are present, THEN patients are more likely to develop minor outcomes (71%)	13
If hypotension occurs, while acidosis, hypoglycemia, electrolyte abnormalities, tachycardia, elevated creatinine, elevated anion gap do not occur, the reason for exposure is unintentional, other miscellaneous are not present, THEN patients are more likely to develop moderate outcomes (75%)	14
If the cause of exposure is unintentional, and acidosis, hypoglycemia, electrolyte abnormalities, tachycardia, elevated creatinine, elevated anion gap and hypotension do not occur, other miscellaneous are not present, patients are more likely to develop minor outcomes (86.5%)	15

**Fig. 3:**
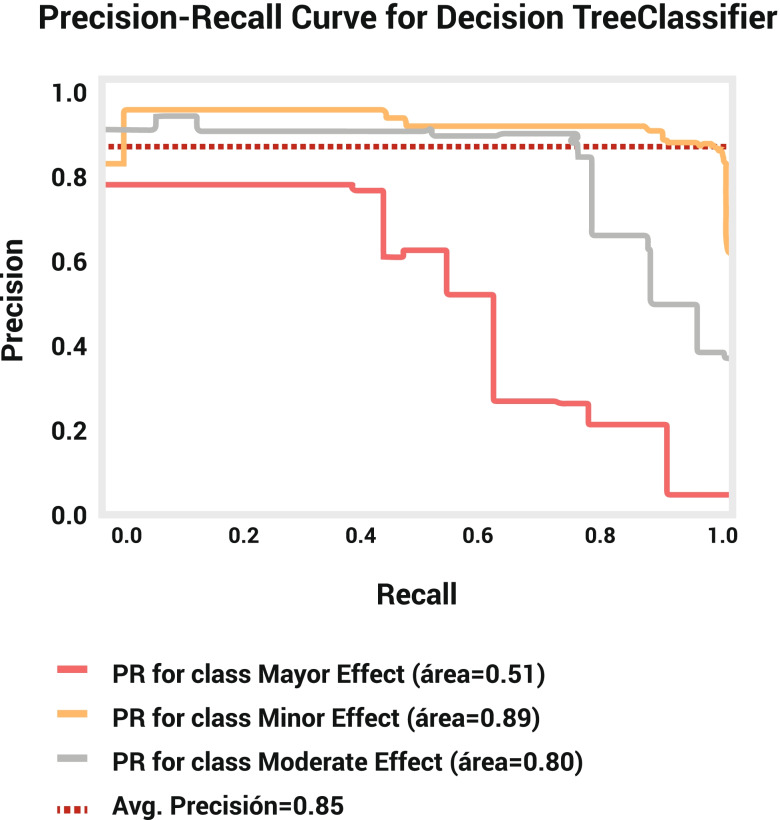
Precision-recall curve for decision tree model

**Fig. 4:**
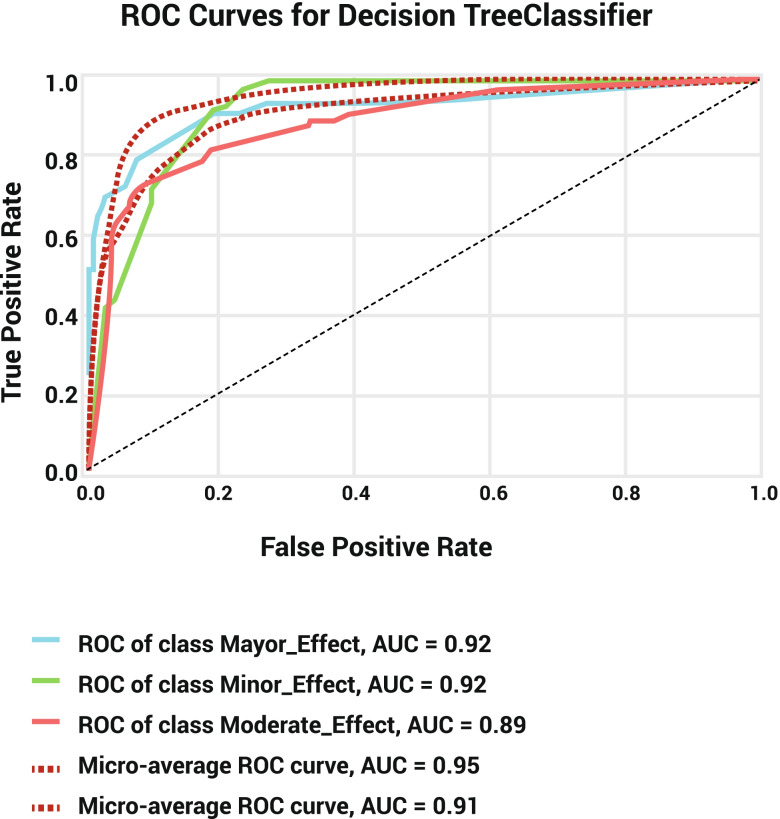
ROC curve for decision tree model

Evaluation of the training and test datasets showed that although the significant levels of recall (training set: 96.42%; test set: 96.54%), precision (training set: 88.16%; test set: 86.21%), and F1-score (training set: 92.10%; test set: 91.08%) attributed to minor outcomes, while the greatest specificity (training set: 99.01%; test set: 98.37%) and belonged to major outcomes (Table 2). This evaluation also demonstrated 87.07 and 85.27% accuracy for training and test datasets. The confusion matrix showed that our model successfully identifies 1266 cases of minor outcomes, 529 cases of moderate outcomes, and 84 cases of major outcomes in the training set and 419 cases of minor outcomes, 167 cases of moderate outcomes, and 28 cases of major outcomes in test datasets (Table 3). The decision tree model’s negative predictive value (NPV) for average, Major, Moderate, and Minor outcomes is 92.30, 97.79, 85.52, and 93.58, respectively. The AUC of the precision-recall curve for major, minor, and moderate outcomes was 0.51, 0.89, and 0.80, respectively (Fig. 3). The AUC of the ROC model for major, minor, and moderate outcomes was 0.92, 0.92, and 0.89, respectively (Fig. 4).

**Table 2:** Characteristics for the training and test sets of SVM and DT

Labels	Dataset	Model	Major effect	Minor effect	Moderate effect	Average	Weighted average
**Specificity**	Training Set	DT	0.990123	0.798817	0.938451	0.909130	0.856677
		SVM	0.999506	0.784615	0.961272	0.915131	0.856145
	Test Set	DT	0.983752	0.765734	0.941300	0.896929	0.838008
		SVM	0.986706	0.786713	0.947589	0.907003	0.852953
**Precision**	Training Set	DT	0.807692	0.881616	0.855987	0.848432	0.868604
		SVM	0.989247	0.876275	0.905724	0.923749	0.892954
	Test Set	DT	0.717949	0.862140	0.856410	0.812166	0.851595
		SVM	0.750000	0.874227	0.874372	0.832866	0.866857
**Recall**	Training Set	DT	0.631579	0.964204	0.742978	0.779587	0.870714
		SVM	0.691729	0.981721	0.755618	0.809690	0.889249
	Test Set	DT	0.651163	0.965438	0.687243	0.767948	0.852778
		SVM	0.627907	0.976959	0.716049	0.773638	0.868056
**F1_score**	Training Set	DT	0.708861	0.921062	0.795489	0.808471	0.866553
		SVM	0.814159	0.926006	0.823890	0.854685	0.885421
	Test Set	DT	0.682927	0.910870	0.762557	0.785451	0.847201
		SVM	0.683544	0.922742	0.787330	0.797872	0.862755
**Accuracy**	Training Set	DT	NaN	NaN	NaN	0.870714	0.870714
		SVM	NaN	NaN	NaN	0.889249	0.889249
	Test Set	DT	NaN	NaN	NaN	0.852778	0.852778
		SVM	NaN	NaN	NaN	0.868056	0.868056

**Table 3:** Confusion matrix for DT and SVM models in the training and test sets

True Prediction	Dataset	Model	Major effect	Minor effect	Moderate effect
**Major effect**	Training Set	DT	84	6	43
		SVM	92	8	33
	Test Set	DT	28	2	13
		SVM	27	1	15
**Minor effect**	Training Set	DT	1	1266	46
		SVM	1	1289	23
	Test Set	DT	0	419	15
		SVM	0	424	10
**Moderate effect**	Training Set	DT	19	164	529
		SVM	0	174	538
	Test Set	DT	11	65	167
		SVM	9	60	174

### Prognosis prediction based on support vector machine

**Fig. 5:**
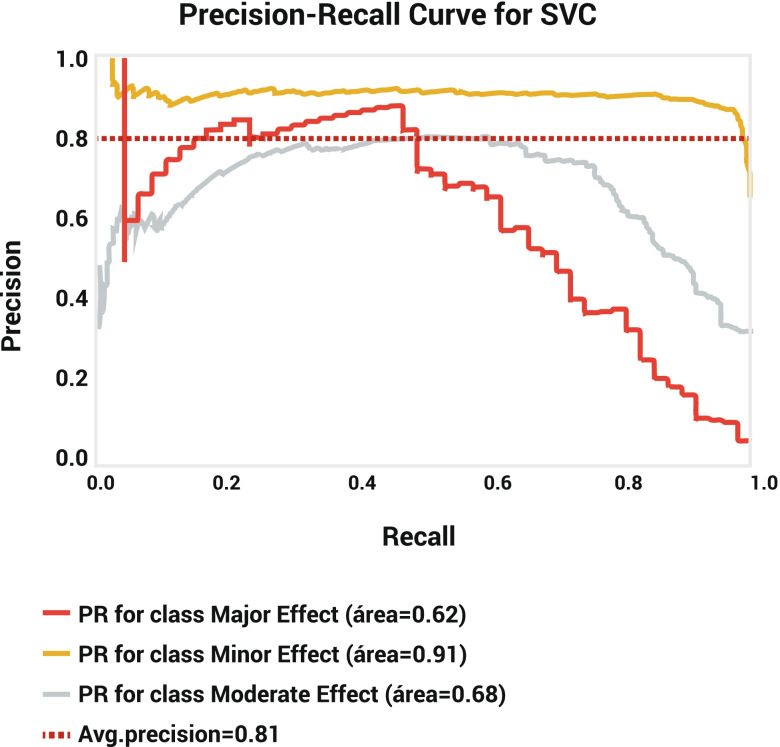
Precision-recall curve for SVM model

**Fig. 6:**
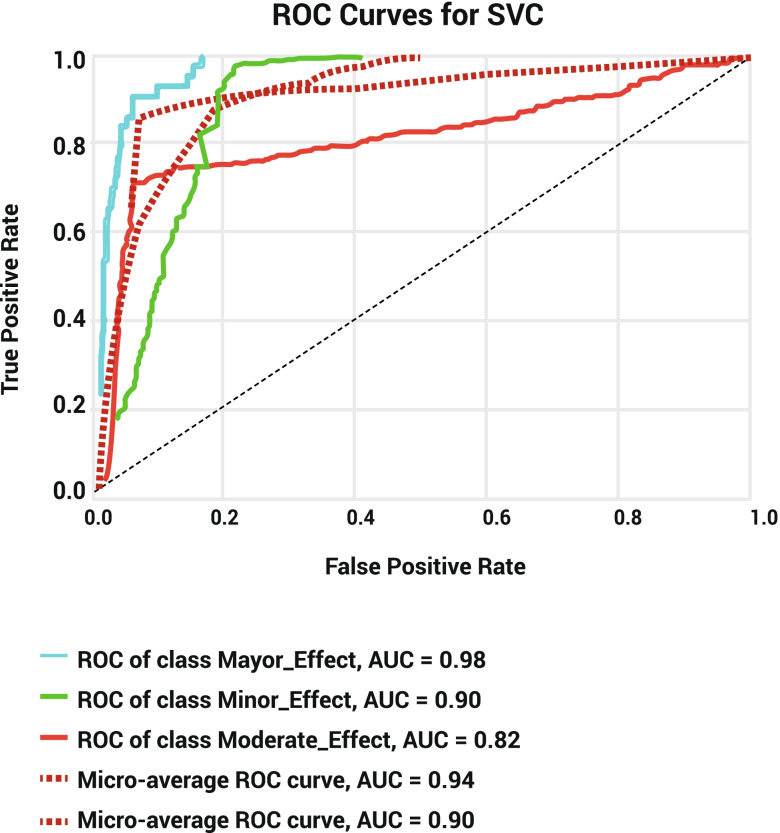
ROC curve for SVM model

Evaluation of the training dataset of the SVM model showed that, despite the greatest specificity of major outcomes in both datasets (training set: 99.95%; test set: 98.67%) as well as the precision in the training set (98.92%), minor outcomes had the greatest recall (training set: 98.17%; test set: 97.69%) and F-1 score (training set: 92.60%; test set: 92.27%) in both datasets. The precision of test sets showed 87.42 and 87.43% for minor and major outcomes, respectively (Table 2). The training and test sets’ accuracy was 88.92 and 86.80%, respectively. The confusion matrix showed that the SVM model could successfully identify 1289 cases of minor outcomes, 538 cases of moderate outcomes, 92 cases of major outcomes in the training set and 424 cases of minor outcomes, 174 cases of moderate outcomes, and 27 cases of major outcomes in test datasets (Table 3). The SVM model’s negative predictive value (NPV) for average, Major, Moderate, and Minor outcomes is 93.30, 97.22, 87.06, and 95.62, respectively. The AUC of the precision-recall curve for major, minor, and moderate outcomes was 0.62, 0.91, and 0.68, respectively (Fig. 5). The AUC of the ROC model for major, minor, and moderate outcomes was 0.98, 0.90, and 0.82, respectively (Fig. 6).

## Discussion

This study is a retrospective cohort study of NPDS data to propose an effective prediction approach to metformin poisoning outcomes. To the best of our knowledge, this is the first study to implement classification approaches in the outcome prediction of metformin poisoning. The current study showed that both DT and SVM algorithms are powerful tools for predicting metformin poisoning outcomes. Moreover, the SVM method predicted the prognosis of metformin poisoning more precisely than the decision tree.

The decision tree and the support vector machine are two of the most well-known machine learning techniques in medicine for uncovering unexplored areas of treatment, prognosis prediction, and diagnosis [21]. One of the important implications of the decision tree is establishing the intervention for groups of people of varying risk categories because there is a prejudice in selecting high-risk groups to define medical guidelines [22]. On the other hand, support vector machines obtain great accuracy when expressing the relationship between multiple elements via a linear feature [23].

In line with other studies, we also showed that acidosis, hypoglycemia, electrolyte abnormality, hypotension, elevated anion gap, elevated creatinine, tachycardia**,** renal failure, age, and unintentional exposure to metformin are the determinants of metformin poisoning prognosis. As we expected, acidosis and hypoglycemia are the most important factors in determining the outcome. Metformin-associated lactic acidosis (MALA) is a fatal condition following metformin poisoning and is accompanied by other gastrointestinal symptoms [24]. MALA has been reported to occur at up to 138 per 100,000 patients per year [25]. It should be noted that early diagnosis of MALA is critical, and it should be suspected in patients with creatinine concentrations of 256 mol/l or higher and lactate concentrations of 8.4 mmol/l or higher [26]. MALA pathogenesis is attributed to a mitochondria blockage of the complex 1 respiratory chain [27]. In addition, lactic acidosis can cause an increase in anion production, which can lead to an increase in the anion gap [28].

Moreover, lactic acidosis is related to gastrointestinal loss, leading to hypovolemia, renal failure, and increased creatinine secondary to lactic acidosis, which can be recognized following metformin overdose [29]. We believe that this hypovolemia might cause hypotension and compensatory tachycardia. Metformin exposure can also reduce glucose absorption, lower hepatic glucose synthesis, and induce hypoglycemia [30].

In our study, we found that the accuracy of the SVM model in predicting the prognosis of metformin poisoning was higher than the DT model. The rationale behind SVM classification is finding the decision boundary to separate different classes and maximize the margin [31]. As a result, this methodology performs well when the sample size is less than the dimensions. However, if additional dimensions are required, it is critical to include other factors such as the kernel and C function to develop a suitable model [12]. Furthermore, even if the data preparation and interpretation are comprehensible in the decision tree model, it cannot deal with the missing non-leaf node [32].

The strength of our study is that we used broad-scale data and introduced a classification approach with high accuracy that can be used in clinical practice. However, some limitations should be mentioned. Data collection of NPDS is based on self-reported cases, meaning that every exposure reported to the NPDS might not be a case of poisoning or overdose. Even though NPDS is the largest database of poisoning in the United States, it does not reflect all of the exposure in the country. While many of the important features in metformin poisoning are known, the most important strength of this study is applying machine learning methods to bring and use these features in clinical practice. This study is a benchmark for other studies in this regard.

## Conclusion

It is important to note that outcome prediction plays a crucial role in managing metformin poisoning. Our ML models to predict metformin poisoning outcomes were very accurate, encouraging clinicians to use these algorithms in clinical practice. In line with other studies, our study also showed that acidosis, hypoglycemia, electrolyte abnormality, hypotension, elevated anion gap, elevated creatinine, tachycardia**,** and renal failure are the most important features in determining the prognosis of metformin. Besides, we found that the support vector model performs more accurately than the decision tree to predict the prognosis. These ML classification models organize our knowledge about metformin poisoning and illustrate the relationship of the important features that can lead to practical algorithms to predict the prognosis of metformin poisoning. Therefore, using machine learning algorithms in determining metformin poisoning is recommended.

## Data Availability

The datasets analyzed during the current study are not publicly available due to patients’ privacy but are available from the corresponding author.
