# Correction: Utility of support vector machine and decision tree to identify the prognosis of metformin poisoning in the United States: analysis of National Poisoning Data System

**DOI:** 10.1186/s40360-022-00608-z

**Published:** 2022-09-09

**Authors:** Omid Mehrpour, Farhad Saeedi, Christopher Hoyte, Foster Goss, Farshad M. Shirazi

**Affiliations:** 1 Data Science Institute, Southern Methodist University, Dallas, TX USA; 2 Rocky Mountain Poison & Drug Safety, Denver Health and Hospital Authority, Denver, CO USA; 3 Medical Toxicology and Drug Abuse Research Center (MTDRC, Birjand University of Medical Sciences (BUMS), Birjand, Iran; 4 Student Research Committee, Birjand University of Medical Sciences, Birjand, Iran; 5 University of Colorado Anschutz Medical Campus, Aurora, CO USA; 6 University of Colorado Hospital, Aurora, CO USA; 7 Department of Emergency Medicine, University of Colorado Hospital, Aurora, CO USA; 8 Arizona Poison & Drug Information Center, the University of Arizona, College of Pharmacy and University of Arizona, College of Medicine, Tucson, AZ USA

**Correction: BMC Pharmacol Toxicol 23, 49 (2022)**

**https://doi.org/10.1186/s40360-022-00588-0**

Following publication of the original article [1], the authors identified some errors in Figs. 1, 2, 3, 4, 5 and 6. The correct figures are given below.

**Fig. 1:**
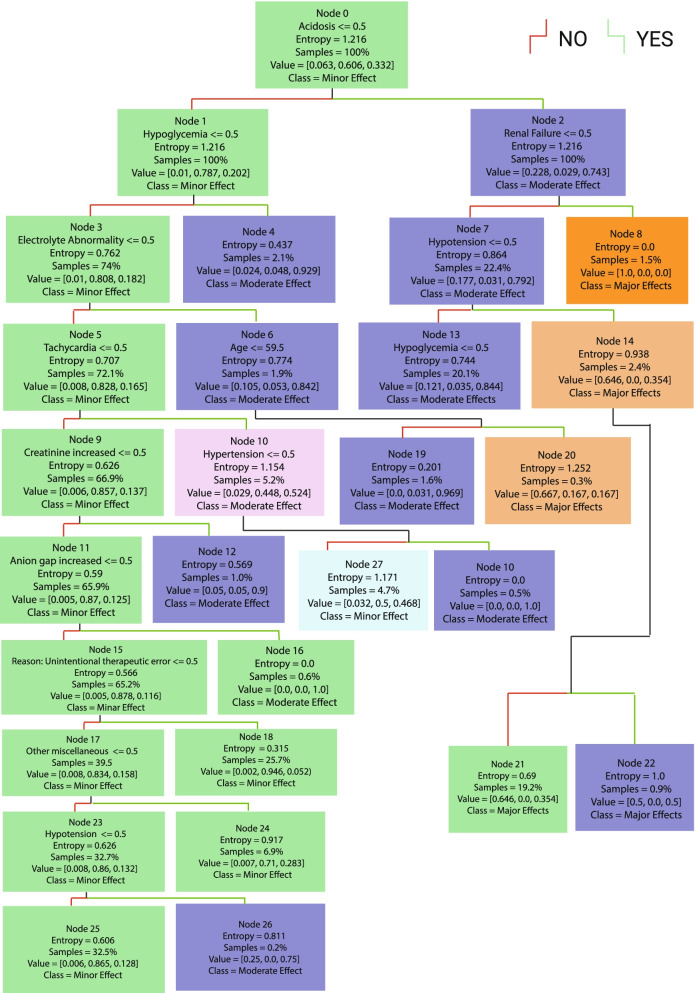
Decision Tree Model1

**Fig. 2:**
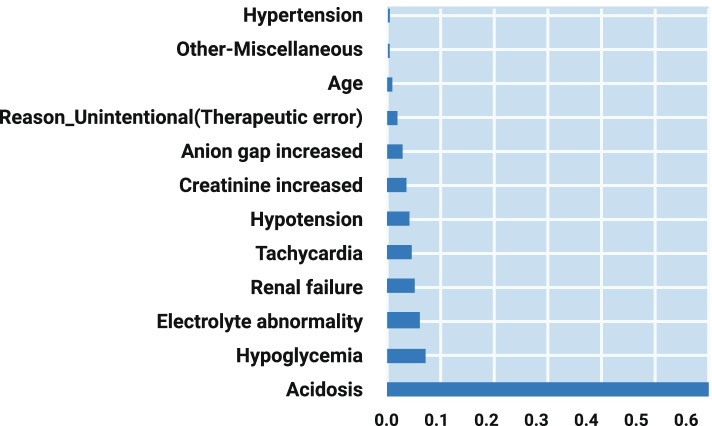
Important Features Based on Decision Tree Algorithm

**Fig. 3:**
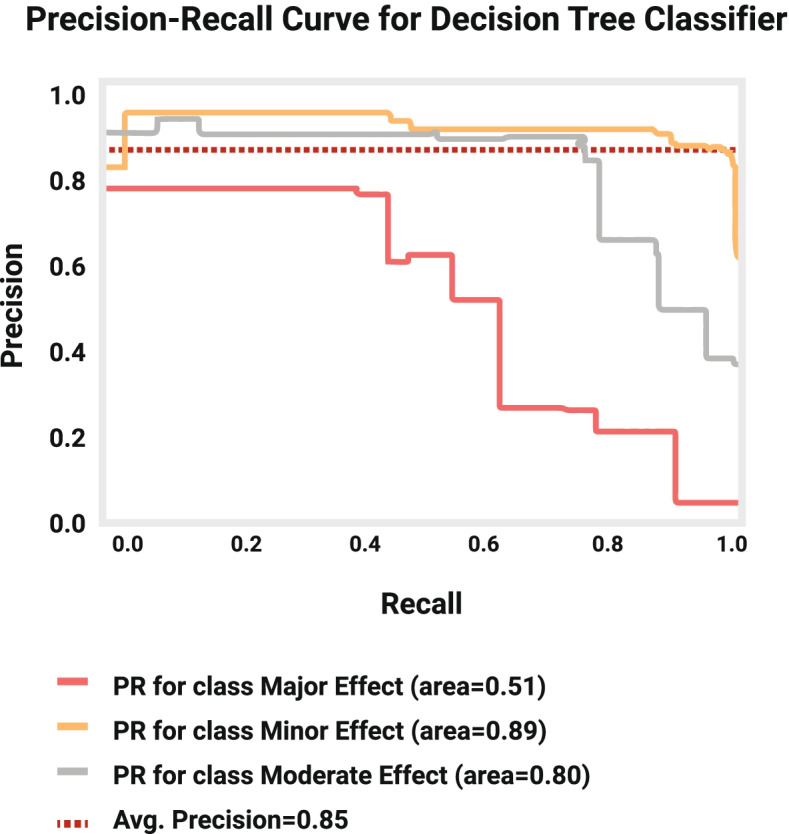
Precision-Recall Curve for Decision Tree model

**Fig. 4:**
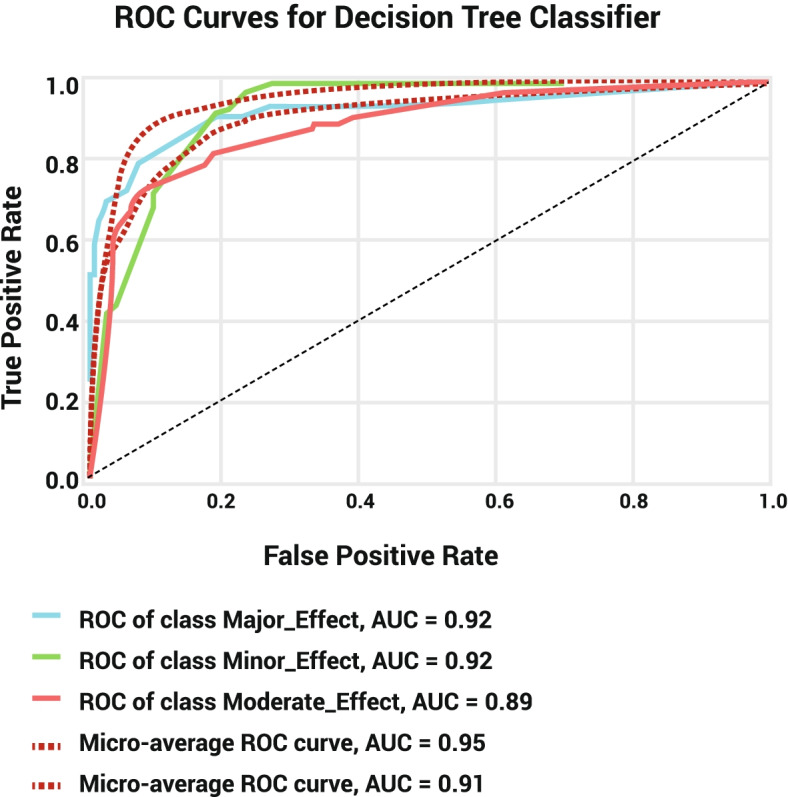
ROC Curve for Decision Tree model

**Fig. 5:**
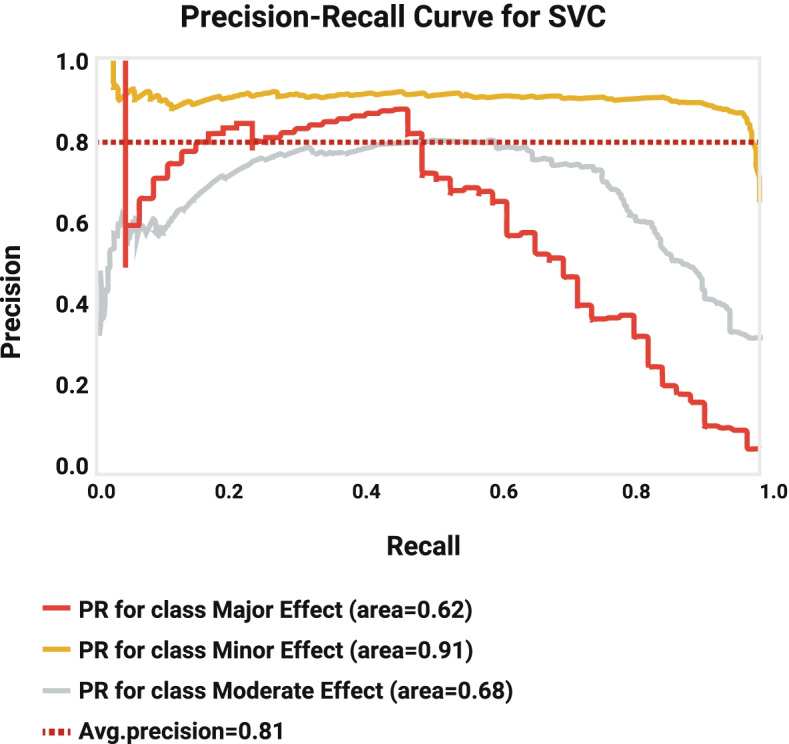
Precision-Recall Curve for SVM model

**Fig. 6:**
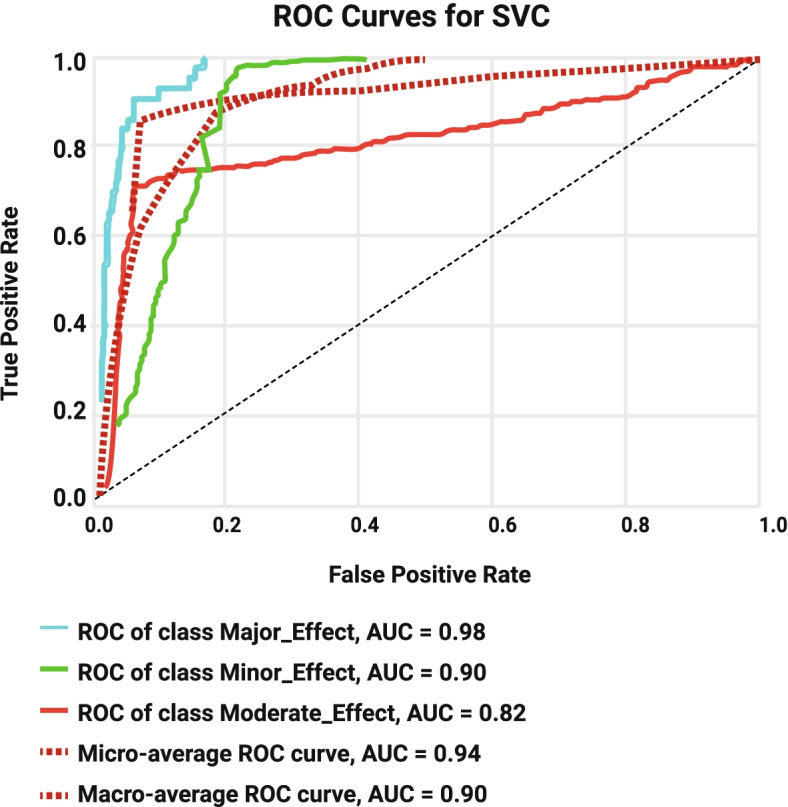
ROC Curve for SVM modell

The original article [1] has been corrected.
